# *Atlanta ariejansseni*, a new species of shelled heteropod from the Southern Subtropical Convergence Zone (Gastropoda, Pterotracheoidea)

**DOI:** 10.3897/zookeys.604.8976

**Published:** 2016-07-11

**Authors:** Deborah Wall-Palmer, Alice K. Burridge, Katja T.C.A. Peijnenburg

**Affiliations:** 1 School of Geography, Earth and Environmental Sciences, Plymouth University, Drake Circus, Plymouth, PL4 8AA, UK; 2 Naturalis Biodiversity Center, Darwinweg 2, 2333 CR Leiden, The Netherlands; 3 Institute for Biodiversity and Ecosystem Dynamics (IBED), University of Amsterdam, P. O. Box 94248, 1090 GE Amsterdam, The Netherlands

**Keywords:** Atlantidae, biogeography, DNA barcoding, shelled heteropod, southern subtropical convergence zone

## Abstract

The Atlantidae (shelled heteropods) is a family of microscopic aragonite shelled holoplanktonic gastropods with a wide biogeographical distribution in tropical, sub-tropical and temperate waters. The aragonite shell and surface ocean habitat of the atlantids makes them particularly susceptible to ocean acidification and ocean warming, and atlantids are likely to be useful indicators of these changes. However, we still lack fundamental information on their taxonomy and biogeography, which is essential for monitoring the effects of a changing ocean. Integrated morphological and molecular approaches to taxonomy have been employed to improve the assessment of species boundaries, which give a more accurate picture of species distributions. Here a new species of atlantid heteropod is described based on shell morphology, DNA barcoding of the Cytochrome Oxidase I gene, and biogeography. All specimens of *Atlanta ariejansseni* **sp. n.** were collected from the Southern Subtropical Convergence Zone of the Atlantic and Indo-Pacific oceans suggesting that this species has a very narrow latitudinal distribution (37–48°S). *Atlanta ariejansseni* **sp. n.** was found to be relatively abundant (up to 2.3 specimens per 1000 m^3^ water) within this narrow latitudinal range, implying that this species has adapted to the specific conditions of the Southern Subtropical Convergence Zone and has a high tolerance to the varying ocean parameters in this region.

## Introduction

The Southern Ocean Sub-Tropical Front (STF) is the boundary between the colder, fresher Sub-Antarctic Zone (SAZ) and the warmer, more saline subtropical waters to the north (Orsi et al. 1995). The Southern Subtropical Convergence Zone (SSTC) is a narrow region along the STF with highly variable physical parameters experiencing strong currents and large gradients of salinity and temperature (Longhurst 1998, Graham and Boer 2013). The STF acts as a dispersal barrier for many zooplankton taxa, resulting in changes in genetic population structure and biomass across this front (Labat et al. 2001, Hiral et al. 2015, Burridge et al. in review a, b). This region is also at a high risk from ocean changes, particularly ocean acidification, because of the high solubility of CO_2_ in cold water (Roberts et al. 2014).

The shelled atlantid heteropods are likely to be particularly susceptible to ocean acidification. Although, to date, there have been no studies into the effects of ocean changes upon atlantids, we can expect that they will react in a similar way to the shelled pteropods (Thecosomata). While not closely related, atlantids share many of the characteristic features that make shelled pteropods vulnerable to ocean acidification. These include living in the upper layers of the ocean, one of the areas most affected, and producing a very small (up to ~10 mm), thin shell of aragonite, which is particularly vulnerable to dissolution in waters undersaturated with carbonate (Fabry et al. 2008). In pteropods, synergistic effects of decreasing carbonate saturation and increasing temperature has been shown to reduce the ability to produce aragonite shells (e.g. Lischka and Riebesell 2012). These effects have already been recorded in natural populations living at high latitudes (Bednaršek et al. 2012), which are predicted to be affected first (Steinacher et al. 2009). However, improvements in taxonomy are extremely important to understanding the effects of these changes on holoplanktonic gastropods. Roberts et al. (2014) found that different forms of the pteropod species *Limacina helicina* (Phipps, 1774), living in the same area of the Southern Ocean, showed opposing trends in shell weight over a long-term study. This demonstrates the importance of assessing species boundaries in order to fully understand the effects of a changing ocean.

Here an integrated morphological and molecular approach is used to present a new species of atlantid heteropod, *Atlanta ariejansseni*, that is restricted to a narrow transitional zone of only 11° of latitude within the SSTC, but has a circumpolar longitudinal range. In common with other sub-polar planktonic gastropod species, *Atlanta ariejansseni* reaches relatively high abundances compared to other atlantids and is the dominant atlantid species living in this area. Most atlantid species are thought to be restricted to warmer tropical and sub-tropical waters, with only one other species, *Atlanta californiensis* Seapy & Richter, 1993, showing a preference for cold water regions in the California Current. *Atlanta ariejansseni* is the only atlantid species specific to sub-polar waters and that appears to be tolerant of such a variable environment.

## Methods

**Figure 1.**
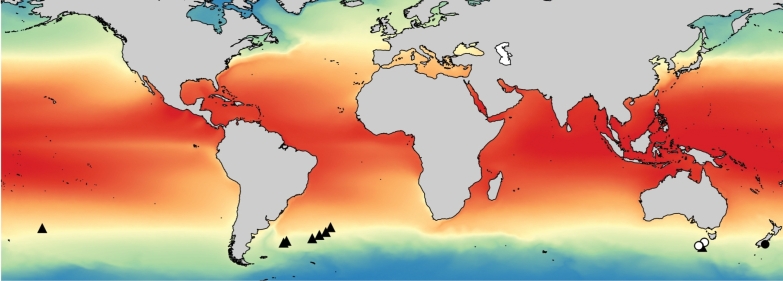
The biogeography of *Atlanta ariejansseni* based on known specimens. Dashed lines show the latitudinal limits of distribution at 37°S and 48°S.

All specimens examined and included in this study were recorded within the SSTC, between 37°S and 48°S (Fig. 1). A total of 184 specimens of *Atlanta ariejansseni* were examined from a number of sources (Table 1). From the Atlantic Ocean, 164 specimens for combined molecular and morphological analysis were collected during the Atlantic Meridional Transects AMT20 and AMT24 (Burridge et al. in review a). On both cruises, specimens were caught using a WP2 bongo net with an aperture diameter of 0.71 m and a mesh of 200 μm. Specimens from AMT24 were fixed and preserved in 96% ethanol and stored at -20 °C prior to DNA barcoding. Specimens from AMT20 were fixed and stored in 96% ethanol and stored at room temperature. Storage at room temperature is not optimal for the preservation of DNA; therefore, specimens from AMT20 were not used for DNA barcoding. From the Pacific Ocean, two further specimens, collected by Erica Goetze during the DRFT cruise of the RV *Revelle* in 2001, were used for molecular analysis (Table 1). Finally, 18 Indo-Pacific specimens were examined from sediment trap samples, collected from south of Tasmania between 1997–2006 by the Antarctic Climate and Ecosystems Cooperative Research Centre (Bray et al. 2000, Roberts et al. 2011). Upon removal from the sediment traps, specimens were washed in buffered peroxide to remove organic matter and dried.

**Table 1.** Details of all known specimens of *Atlanta ariejansseni*, including sampling information.

Ocean	Cruise or project	Station	Latitude	Longitude	Sampling depth	Sampling time (local)	SST (°C)	Bottom depth (m)	Type of material	No. specimens		Notes on specimen use and storage	Institute or reference
										Adult	Juvenile		
Atlantic	AMT20	33	-44,20	-48,95	200	04:25–05:32	-	5223	Plankton haul specimens in ethanol.	3	0	2 paratypes coated for SEM. 1 specimen destroyed for radula extraction.	Plymouth Marine Laboratory
		74	-45,02	-50,28	-	13:09–14:03	-	5695		0	10	4 paratypes, 2 coated for SEM, 2 in 96% ethanol. 6 specimens in 96% ethanol.	
	AMT24	26	-37,89	-28,74	372	03:04–03:54	13,68	3622		1	2	2 specimens DNA barcoded (juvenile, destroyed). 1 remaining in 96% ethanol.	Naturalis Biodiversity Center
		27	-40,12	-30,91	216	03:03–03:52	13,89	4491		8	13	5 specimens DNA barcoded (3 adult, 2 juvenile, destroyed). Remaining specimens in 96% ethanol.	
		28	-41,48	-33,86	228	02:59–03:48	11,5	4943		8	69	1 holotype in 96% ethanol (adult). 1 specimen DNA barcoded (adult, destroyed). Remaining specimens in 96% ethanol.	
		29	-43,02	-37,14	253	03:00–03:49	11,5	5219		13	35	7 specimens DNA barcoded (4 adult, 3 juvenile, destroyed). Remaining specimens in 96% ethanol.	
Pacific	DRFT	14	-38,32	-161,14	-	-	-	-		1	1	2 specimens DNA barcoded (destroyed).	
	n/a	Taiaroa Head	-45,77	170,89	-	-	-	-	Plankton hauls, published data	Abundant		n/a	Pilkington (1970)
Indo-Pacific	SAZ-Sense	47°S	-47,00	141,00	-	-	-	-		1			Howard et al. 2011
		TS-2	-44,88	142,98	20	1:12	-	-		4			
		PS-1 V. haul	-46,42	140,53	20	13:55–10:25	-	-		7			
		PS-1 RMT 1	-46,47	140,37	30-70	18:37–19.11	-	-		2			
		47°S	-47,76	142,07	-	-	-	-	Dry shells, sediment trap	2	16	2 paratypes (J). All specimens dry.	Bray et al. 2000

Two published records of atlantids are also available for this region and both are considered here to include misidentified specimens of *Atlanta ariejansseni* sp. n. Howard et al. (2011) recorded 14 specimens of *Atlanta gaudichaudi* Gray, 1850 in net hauls and a sediment trap positioned south of Tasmania. However, specimens from the same sediment traps (Roberts et al. 2011) that were re-examined for this study were also originally misidentified as *Atlanta gaudichaudi*. A single image of a specimen caught by Howard et al. (2011) is morphologically consistent with *Atlanta ariejansseni*, but is too small to identify with certainty.

Pilkington (1970) described a single species of atlantid, provisionally identified as *Atlanta helicinoidea* Gray, 1850, off-shore of Taiaroa Head, New Zealand. Pilkington (1970) found it difficult to identify specimens to species level, noting that the morphology did not agree perfectly with any of the atlantid species that had already been described. The detailed descriptions and figures presented by Pilkington (1970) unquestionably resemble the shell morphology of *Atlanta ariejansseni*. Moreover, descriptions of the juvenile stages made by Pilkington (1970) match the juvenile specimens that were examined for this study. Therefore, the *Atlanta* specimens described by Pilkington (1970) are considered to be *Atlanta ariejansseni*.

### DNA barcoding

A total of 17 undamaged adult (N = 9) and juvenile (N = 8) specimens of *Atlanta ariejansseni* were selected from samples collected during AMT24 and DRFT research cruises. DNA barcoding was also carried out for the morphologically similar species *Atlanta selvagensis* de Vera & Seapy, 2006 from the Atlantic Ocean. Five specimens of adult (N = 2) and juvenile (N = 3) *Atlanta selvagensis* were selected from AMT24 sites (St. 5, 34.75°N, 26.62°W; St. 6, 31.30°N, 27.73°W and St. 14, 3.8°N, 25.78°W). All specimens were imaged prior to analysis using a Zeiss automated z-stage light microscope. DNA was extracted from whole specimens, using the NucleoMag 96 Tissue kit by Macherey-Nagel on a Thermo Scientific KingFisher Flex magnetic bead extraction robot, with a final elution volume of 75 μl. A standard Cytochrome Oxidase I (COI) barcoding fragment (Hebert et al. 2003) was amplified using primers jgLCO1490 and jgHCO2198 (Geller et al. 2013). Primers were tailed with M13F and M13R for sequencing (Messing 1983). PCR reactions contained 17.75 μl mQ, 2.5 μl 10x PCR buffer CL, 0.5 μl 25mM MgCl_2_, 0.5 μl 100mM BSA, 1.0 μl 10 mM of each primer, 0.5 μl 2.5 mM dNTPs and 0.25 μl 5U Qiagen Taq, with 1.0 μl of template DNA, which was diluted 10 or 100 times for some samples. PCR was performed using an initial denaturation step of 180 s at 94 °C, followed by 40 cycles of 15 s at 94 °C, 30 s at 50 °C and 40 s at 72 °C, and finishing with a final extension of 300 s at 72 °C and pause at 12 °C. Sequencing was carried out by Macrogen, Europe.

**Figure 2.**
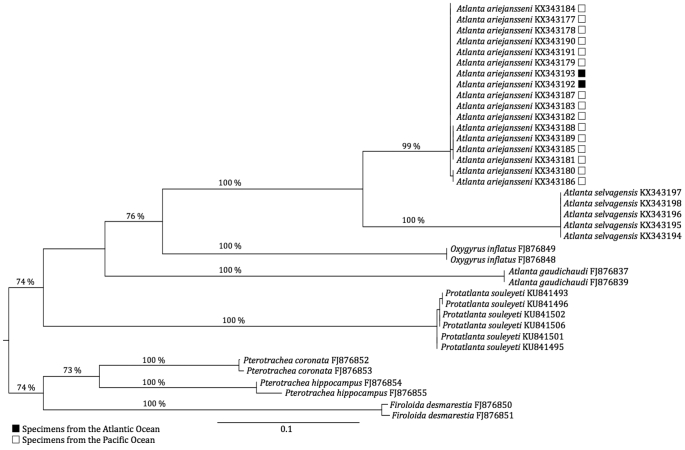
Maximum-likelihood tree showing the relationship of *Atlanta ariejansseni* to different species of *Atlanta*, different Atlantidae genera, and different Pterotracheoidea families, based on Cytochrome Oxidase I DNA sequences. Branch lengths are proportional to the amount of inferred change, indicated by the scale bar. Only bootstrap support (1000 replicates) above 70% are displayed. GenBank sequence numbers are presented in Table 2. Sequences from Jennings et al. 2010 begin with FJ.

All sequences were aligned and edited using the ClustalW algorithm in MEGA 6 (Tamura et al. 2013) and submitted to GenBank (Fig. 2, Table 2). Previously published COI sequences from GenBank (Jennings et al. 2010, Wall-Palmer et al. in press), identified as *Atlanta inclinata* Gray, 1850, *Oxygyrus inflatus* Benson, 1835, *Firoloida desmarestia* Lesueur, 1817, *Pterotrachea hippocampus* Philippi, 1836, *Pterotrachea coronata* Forsskål in Niebuhr, 1775 and *Protatlanta souleyeti* (Smith, 1888), were added to represent the families and genera most closely related to *Atlanta ariejansseni*. Based on these data, a maximum-likelihood tree was constructed in MEGA6 using nucleotide sequences in a General Time Reversible model with gamma distribution and invariant sites (GTR+G+I) and 1000 bootstraps. Kimura-2-parameter (K2P) genetic distances were calculated between and within species belonging to the family Atlantidae using MEGA 6 (Tamura et al. 2013).

**Table 2.** Original specimen codes and GenBank accession numbers for all specimens included in the phylogenetic analysis (Fig. 2).

Species	Specimen code or reference	GenBank accession number
*Atlanta ariejansseni*	Aari_AMT24_26_01	KX343177
	Aari_AMT24_26_02	KX343178
	Aari_AMT24_27_01	KX343179
	Aari_AMT24_27_02	KX343180
	Aari_AMT24_27_03	KX343181
	Aari_AMT24_27_04	KX343182
	Aari_AMT24_27_05	KX343183
	Aari_AMT24_28_01	KX343184
	Aari_AMT24_29_01	KX343185
	Aari_AMT24_29_02	KX343186
	Aari_AMT24_29_03	KX343187
	Aari_AMT24_29_04	KX343188
	Aari_AMT24_29_05	KX343189
	Aari_AMT24_29_06	KX343190
	Aari_AMT24_29_07	KX343191
	Aari_DRFT_14_01	KX343192
	Aari_DRFT_14_02	KX343193
*Atlanta selvagensis*	Asel_AMT24_05_03	KX343194
	Asel_AMT24_06_01	KX343195
	Asel_AMT24_06_02	KX343196
	Asel_AMT24_06_04	KX343197
	Asel_AMT24_14_02	KX343198
*Atlanta gaudichaudi*	Jennings et al. 2010	FJ876837
		FJ876839
*Oxygyrus inflatus*		FJ876848.1
		FJ876849.1
*Protatlanta souleyeti*	Wall-Palmer et al. in press	KU841501
		KU841495
		KU841506
		KU841502
		KU841497
		KU841494
		KU841496
		KU841493
*Pterotrachea coronata*	Jennings et al. 2010	FJ876852.1
		FJ876853.1
*Pterotrachea hippocampus*		FJ876854.1
		FJ876855.1
*Firoloida desmarestia*		FJ876850.1
		FJ876851.1

## Results and discussion

### Genetic diversity

DNA barcoding of seventeen *Atlanta ariejansseni* specimens and five *Atlanta selvagensis* specimens from the southern Atlantic (N = 15, N = 5 respectively) and Pacific (N = 2, N = 0 respectively) oceans shows that *Atlanta ariejansseni* forms a monophyletic group with a bootstrap support of 100% (Fig. 2). *Atlanta ariejansseni* has an average K2P distance of 0.14–0.25 from other species in the genus *Atlanta* and 0.22–0.26 from other genera of Atlantidae (*Oxygyrus* and *Protatlanta* respectively, Table 3).

**Table 3.** Average K2P distances between *Atlanta ariejansseni* and the Atlantidae species *Atlanta gaudichaudi*, *Atlanta selvagensis*, *Protatlanta souleyeti* and *Oxygyrus inflatus*.

	***Atlanta ariejansseni***	***Atlanta gaudichaudi***	***Atlanta selvagensis***	***Protatlanta souleyeti***
*Atlanta ariejansseni* (n = 17)				
*Atlanta gaudichaudi* (n = 2)	0,25			
*Atlanta selvagensis* (n = 5)	0,14	0,27		
*Protatlanta souleyeti* (n = 6)	0,26	0,24	0,24	
*Oxygyrus inflatus* (n = 2)	0,22	0,25	0,25	0,25

### Biogeography

**Figure 3.**
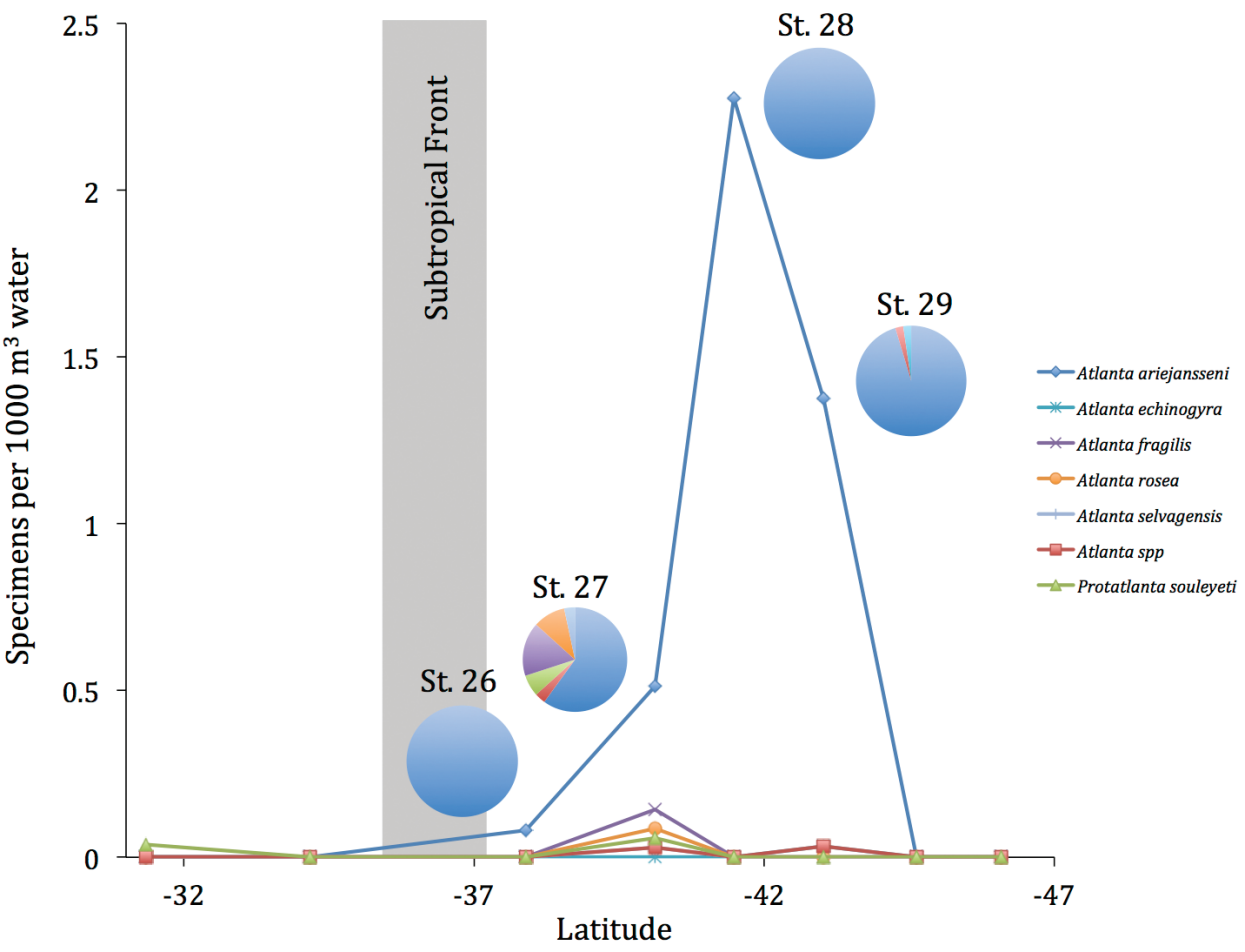
Abundance and pie charts of relative abundance (%) of atlantids at southern Atlantic stations of the AMT24 cruise.

All known specimens of *Atlanta ariejansseni* were collected between 37°S and 48°S (Table 1) within the SSTC in water temperatures of 6.5–14.3°C (Fig. 1). Along the AMT24 transect, the most northern occurrence of the key thecosome pteropod species *Limacina helicina antarctica* Woodward, 1854 was at St. 26 (31.34°S), the same station as *Atlanta ariejansseni* (Burridge et al. in review a). However, the range of *Limacina helicina antarctica* extends much further south than *Atlanta ariejansseni*, which, along with all other atlantid species, were not found at sites south of 48°S. In the Atlantic Ocean, *Atlanta ariejansseni* was found at four AMT24 stations (St. 26–29) between 37°S and 43°S. *Atlanta ariejansseni* was found to be the most abundant atlantid at these stations and the only species present at stations 26 and 28 (Fig. 3). At a latitude of -41.47°S, *Atlanta ariejansseni* reached a maximum abundance of 2.3 specimens per 1000 m^3^.

Specimens of *Atlanta ariejansseni* have been caught at different times of the day in the upper 372 m of the water column (Table 1). Low numbers of specimens were caught at the ocean surface (20–70 m) at all times of the day. However, highest numbers were caught in 228–253 m water depth at night between 03:00 and 04:00 local time (Table 1).

## Systematics

### Phylum MOLLUSCA Class GASTROPODA Cuvier, 1797 Subclass CAENOGASTROPODA Cox, 1960 Order LITTORINIMORPHA Golikov & Starobogatov, 1975 Superfamily PTEROTRACHEOIDEA Rafinesque, 1814 Family ATLANTIDAE Rang, 1829 Genus *Atlanta* Lesueur, 1817

#### Atlanta ariejansseni sp. n.

Taxon classificationAnimaliaLittorinimorphaAtlantidae

http://zoobank.org/7E9AEE5E-5F7F-480C-9673-89A3E9979FE9

Figures 4 , 5 , 6

##### Type locality.

AMT24 station 28, 41.48°S, 33.86°W. Specimen collected on the 27^th^ October 2014 at 02:59–03:48 local time at a water depth of 0–228 m.

##### Holotype.

Figure 5j–l. Housed at the Naturalis Biodiversity Center, Leiden, accession number RMNH.5004155. For specimen dimensions, see Table 4. Collected by Alice K Burridge.

**Table 4.** Overview of type material.

Specimen	Description	Illustrated?	Locality		Institute registration number	Storage	Dimensions	
			Latitude	Longitude			Number of whorls	Diameter without keel (μm)
Aari_AMT24_28_01 (holotype)	Adult	5j–l	-41,48	-33,86	RMNH.5004155	Wet 96% ethanol	4½-4¾	2260
Aari_AMT20_33_01 (paratype)	Adult	Fig. 4a, c–d	-44,20	-48,95	RMNH.5004156	Dry, coated for SEM	4¼−4½	1478
Aari_AMT20_33_02 (paratype)	Adult	Fig. 4b, e–f	-44,20	-48,95	RMNH.5004157	Dry, coated for SEM	4½	2336
Aari_AMT20_74_01 (paratype)	Juvenile	Fig. 4i	-45,02	-50,28	RMNH.5004158	Dry, coated for SEM	3	330
Aari_AMT20_74_02 (paratype)	Juvenile	Fig. 5k	-45,02	-50,28	RMNH.5004159	Dry, coated for SEM	3½	480
Aari_AMT20_74_03 (paratype)	Juvenile	none	-45,02	-50,28	RMNH.5004160	Wet 96% ethanol	-	-
Aari_AMT20_74_04 (paratype)	Juvenile	none	-45,02	-50,28	RMNH.5004161	Wet 96% ethanol	-	-
Aari_47S_01 (paratype)	Juvenile	Fig. 4g	-47,00	141,00	NHMUK 20160080	Dry	3½	460
Aari_47S_02 (paratype)	Juvenile	Fig. 4h	-47,00	141,00	NHMUK 20160081	Dry	3¾	543

##### Paratypes.

**Figure 4.**
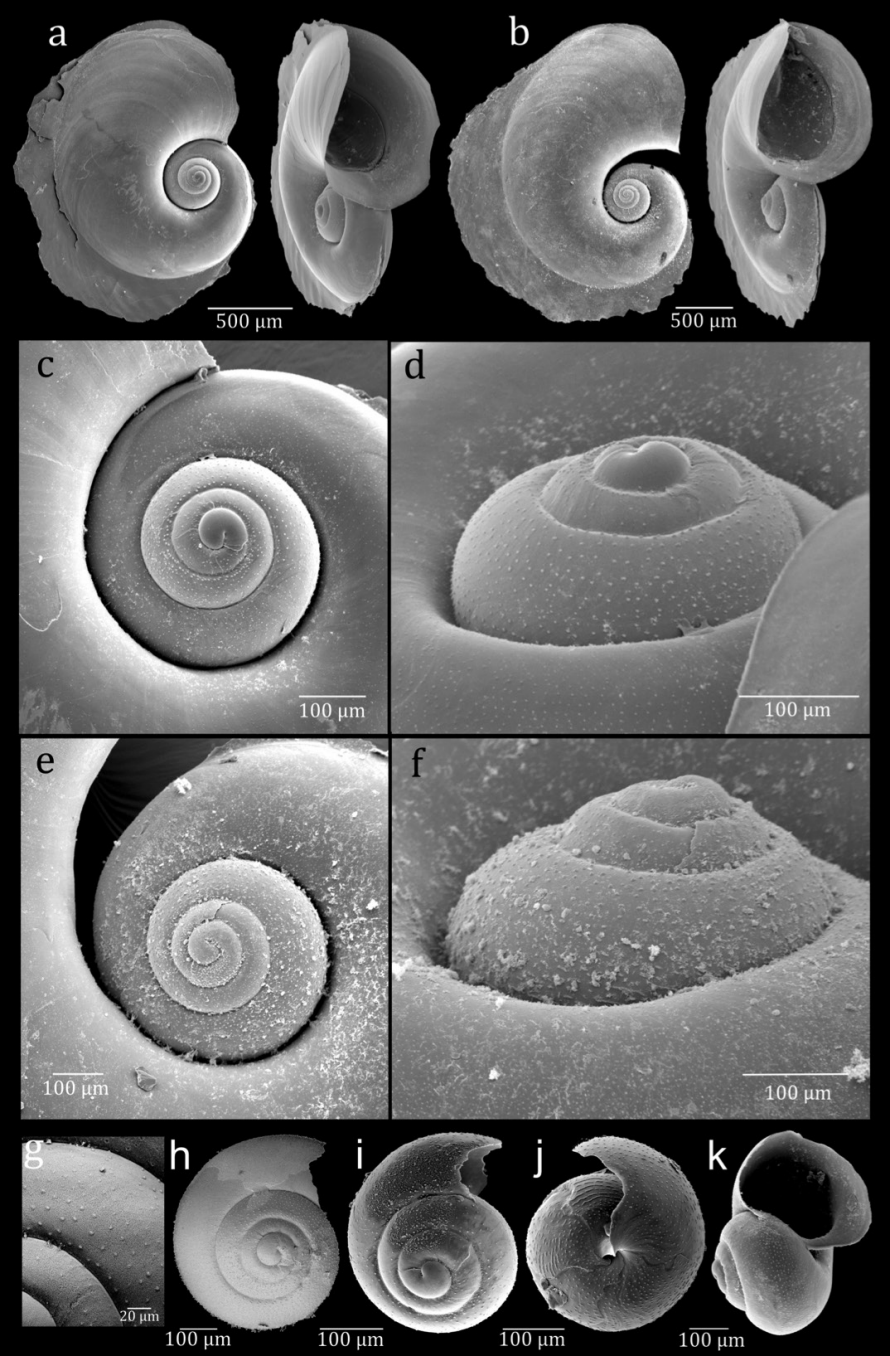
SEM images of *Atlanta ariejansseni*. Aari_AMT20_33_01 (**a, c–d**); Aari_AMT20_33_02 (**b, e–f**); Aari_47S_01 (**g**); Aari_47S_02 (**h**); Aari_AMT20_74_01 (**i**); Aari_AMT20_74_05 (**j**); Aari_AMT20_74_02 (**k**). Specimens g and h were imaged using low vacuum SEM and were not sputter coated.

Figure 4a–i and k. See Table 4 for details.

**Figure 5.**
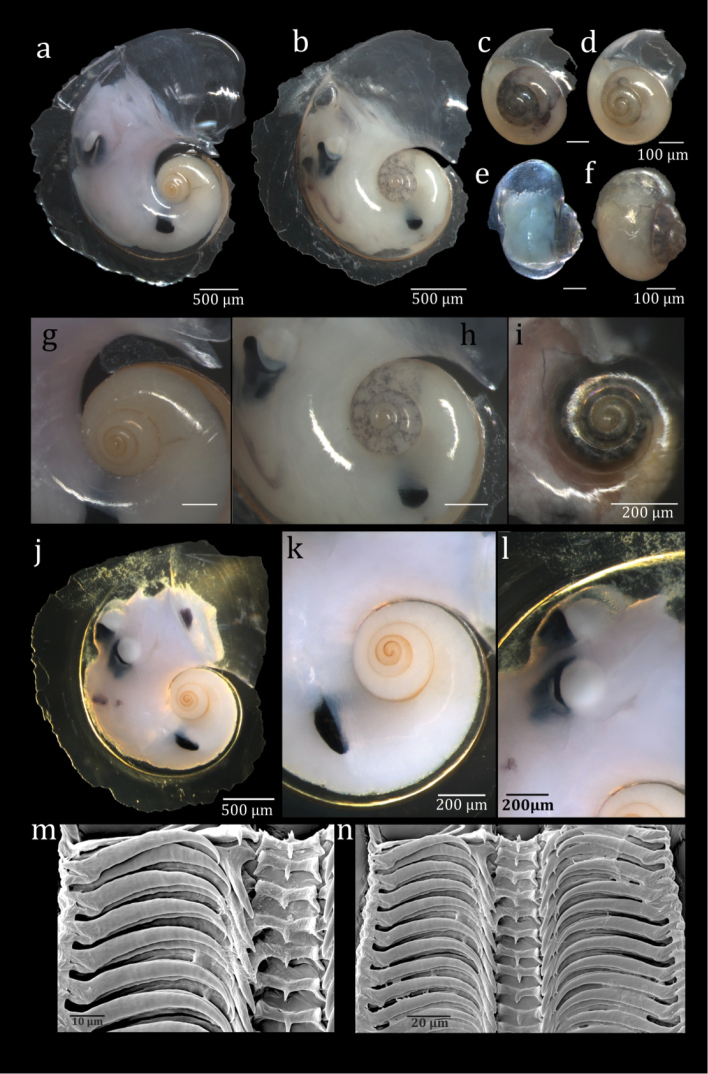
Stacking light microscopy images of *Atlanta ariejansseni* showing variations in tissue colour. Aari_AMT24_29_01 (**a, g**); Aari _AMT24_27_01 (**b, h**); Aari_AMT24_26_01 (**c**); Aari_AMT24_26_02 (**d**); Aari_AMT24_27_04 (**e**); Aari_AMT24_27_04 (**f**); Aari_AMT24_28_01 (**i**); Aari_AMT24_28_01 (**j–l**); Radula of Aari_AMT20_33_03 (**m–n**).

##### Additional material.

See Table 1.

##### Diagnosis.

*Atlanta* species with a spire of 3 ¼ to 3 ½ whorls. The spire is moderately high, rounded and with deep sutures and covered in small, low projections approximately arranged in lines.

##### Description.

Shell small and transparent, with adult shells ranging from 2012 to 3059 μm in diameter excluding the keel and 2237 to 3370 μm including the keel in examined material. The shell inflates at 3 ¼ to 3 ½ whorls and has a total of 4 ½ to 4 ¾ whorls. The keel begins at 3 ¾ whorls and inserts between the final whorl and the spire for around ¼ whorl. The keel is tall and gradually truncated with a yellow-brown keel base. The keel often has a slightly undulating shape. The soft tissue varies greatly in colour among individuals from mottled white to orange-pink and dark grey (Fig. 5). Some specimens were observed to have a pearlescent lustre to the shell surface.

**Figure 6.**
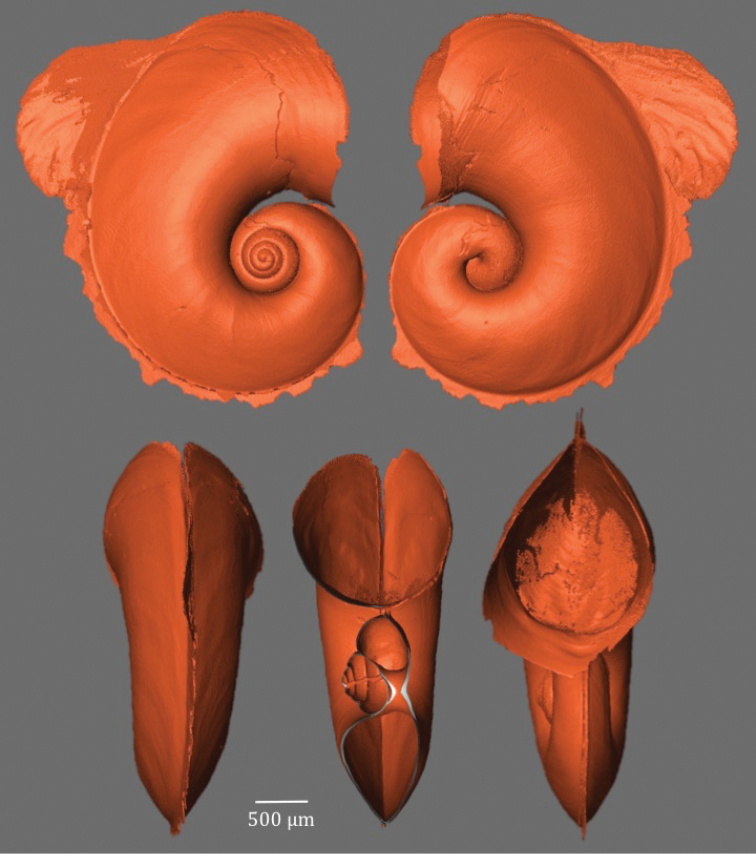
X-ray tomography of *Atlanta ariejansseni* specimen Aari_AMT20_33_03.

The spire is moderately high, well-visible in apertural view, with deep sutures, giving the whorls a rounded appearance (Fig. 6). The spire surface is ornamented with numerous low projections in the form of punctae roughly arranged in 9–12 spiral rows over the surface of whorls 2–4 (Fig. 4). These low projections can vary in their spatial coverage, from closely spaced to sparse (Fig. 4g–h). This gives the spire a rough appearance under a light microscope. The projections are clearly visible using SEM (Fig. 4). No other species of atlantid has been found with this type of micro-ornamentation in the inner spire. Juvenile specimens have approximately six fine lines of small projections running around the side of the shell, although these are not always obvious under light microscopy. Around the base of the juvenile shell the projections can become so closely positioned that they become irregular, frequently interrupted spiral lines in some specimens (Fig. 4j)

The operculum is type c, the radula is type I (Fig. 5m–n) and the eyes are of type a (Seapy et al. 2003), with no transverse slit (Fig. 5h and l).

##### Discussion.

The rounded spire, number whorls, opercular, radula and eye type all suggest that *Atlanta ariejansseni* belongs within the *Atlanta inflata* group of Richter and Seapy (1999). The most morphologically similar species are *Atlanta californiensis* and *Atlanta selvagensis. Atlanta californiensis* has the same number of whorls in the spire and the same overall adult shape as *Atlanta ariejansseni*, but it does not have any shell ornamentation. *Atlanta californiensis* also has much shallower spire sutures than *Atlanta ariejansseni*. *Atlanta selvagensis* is a slightly smaller species that does show shell ornamentation of the spire in the form of spiral lines that are frequently interrupted and highly variable; however, the ornamentation of *Atlanta ariejansseni* can clearly be distinguished from that of *Atlanta selvagensis*. Molecular results presented here also confirm that the two species are closely related, but separated by a K2P genetic distance of 0.14. No molecular data is available for *Atlanta californiensis*.

Previous publications have identified *Atlanta ariejansseni* as *Atlanta gaudichaudi* (Howard et al. 2011) and *Atlanta helicinoidea* (Pilkington 1970). However, these two species are also morphologically different from *Atlanta ariejansseni*. Although *Atlanta helicinoidea* belongs to the *Atlanta inflata* group, the spire has an extra whorl and the ornamentation is much coarser than that of *Atlanta ariejansseni*. *Atlanta gaudichaudi* is described as having no shell ornamentation, although some authors show this species with a single spiral line on the spire (Seapy et al. 2003). However, *Atlanta gaudichaudi* does not have the low projections that are found on the spire of *Atlanta ariejansseni*. DNA barcoding also shows that these two species are not closely related, with an average K2P genetic distance of 0.25.

##### Distribution.

All specimens were found between 37°S and 48°S latitude, in a narrow circumtropical band located in the Southern Subtropical Convergence Zone. Specimens were collected from the epipelagic layer (upper 372 m) using oblique plankton tows in the Atlantic and Pacific oceans. For a summary of biogeography and sampling information, see Fig. 1 and Table 1.

##### Etymology.

Named after Arie Janssen, Naturalis Biodiversity Center, Netherlands, in recognition of his commitment and longstanding contributions to holoplanktonic gastropod research.

## Conclusions

Combined molecular, morphological, and biogeographical information has allowed the introduction of a new species of the genus *Atlanta* that can be easily identified by means of its shell ornamentation using light microscopy. *Atlanta ariejansseni* is the only atlantid species that has been found living at high latitudes, restricted to a narrow circumpolar region. It is, therefore, an extremely important species in the current race to understand the effects of a changing ocean. It can be assumed that this species is able to tolerate a variable environment, which suggests that it may also be able to adapt to a changing ocean. This resilience and adaptability may be demonstrated by the successful rearing of veliger *Atlanta ariejansseni* through to adults under laboratory conditions by Pilkinton (1970), which has never since been accomplished with other atlantid species.

Large sampling efforts have been made for holoplanktonic gastropods in the Southern Ocean; however, *Atlanta ariejansseni* has never been recognised as an undescribed species in these studies. This is undoubtedly due to our incomplete understanding of atlantid taxonomy, particularly for the Atlantic Ocean. We hope that this study will increase awareness of *Atlanta ariejansseni* and encourage others to record this circumpolar species when observed to build up a more complete biogeography. It is only with more biogeographical and ecological data that we will be able to determine the ecology and effects of a changing ocean upon this species.

## Supplementary Material

XML Treatment for Atlanta ariejansseni
